# An interview with Matheus Melo Pithon

**DOI:** 10.1590/2176-9451.20.3.018-028.int

**Published:** 2015

**Figure:**
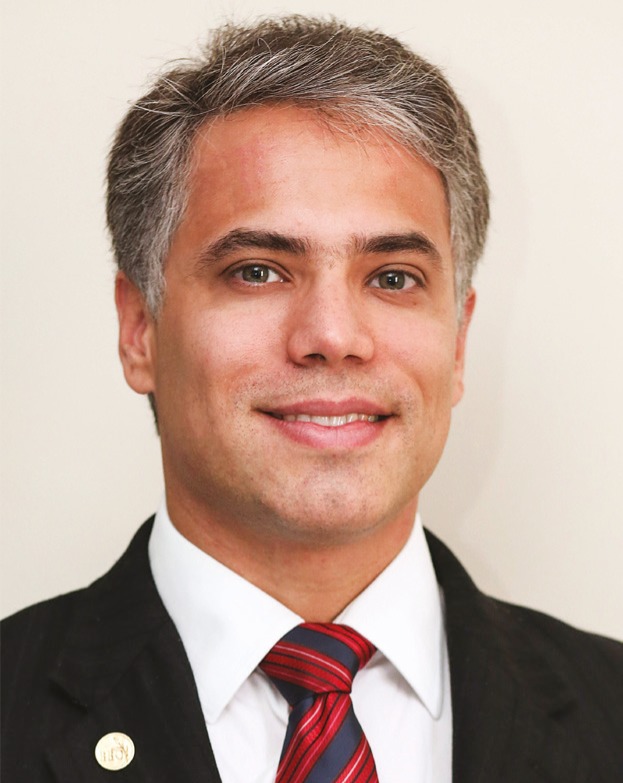

During one of my trips to the Universidade Federal do Rio de Janeiro (UFRJ), as a PhD resident, Dr. Antônio Carlos Ruellas greeted me with his usual kindness and told me: "Let's go to the lab. I would like to introduce you to two new orthodontic residents who graduated from a Dental School at your home state. They are excellent and they write really good papers." One of them was Dr. Matheus M. Pithon. Less than 10 years latter, Dr. Pithon became an orthodontist with great scientific production in Brazil, and certainly one of the most academically productive worldwide. All this academic success was achieved before he turned 40 years old and while he was affiliated to the Universidade Estadual do Sudoeste da Bahia (UESB), located in the heart of Bahia state (Brazil). When I think about what Dr. Pithon has been able to achieve, one word comes to my mind: impressive. His ethics, his ability to motivate people to believe in their work and his unbelievable determination to publish his papers in the best possible scientific journals are remarkable. However, even with all academic success, Dr. Pithon also has a part-time private clinic in Vitória da Conquista (Bahia, Brazil) and has been certified by the Brazilian Board or Orthodontics (BBO) since 2011. Combining excellence in academics and in the private practice has been one of Dr. Pithon's features in his orthodontic career so far. One of his latest papers, which assessed the influence of dental esthetics in finding a job, obtained great visibility in the American Journal of Orthodontics and Dentofacial Orthopedics. It also attracted the attention of Brazilian local news media. Despite his success, Dr. Pithon is a simple and humble person, admired and loved by his students. These personal characteristics may be due to his strong family values. He is married to Ana Carolina, a dermatologist and university professor. They have a one-year-old son, João Pedro. When Dr. Pithon is not consulting patients at his office or teaching and writing papers, he is spending time with his family or enjoying his new passion: raising cattle and horses. On the next few pages, Dr. Pithon will tell us about his short, but rather intense career in Orthodontics, either as a clinician, a researcher or a professor. (Dauro Douglas Oliveira)

Em uma de minhas idas à UFRJ, como doutorando em Ortodontia (2006), o Prof. Antônio Carlos Ruellas, após receber-me com a costumeira atenção, disse: "*Vamos ao laboratório, pois quero apresentá-lo a dois novos mestrandos que fizeram graduação em Minas Gerais. Eles são ótimos alunos e escrevem artigos muito bem.*" Um deles era o Dr. Matheus Pithon. Menos de 10 anos depois, o Dr. Matheus se tornou o ortodontista com maior produção científica do Brasil e, certamente, um dos mais produtivos do mundo. Isso antes de completar 40 anos de idade, e lecionando no interior da Bahia, em uma universidade sem programas de pós-graduação na sua instituição. Quando paro e penso no que o Prof. Matheus tem conseguido fazer, a primeira palavra que me vem à cabeça é: impressionante. Sua capacidade de trabalho, de motivar e mobilizar alunos do curso de graduação, e sua incrível determinação em gerar artigos científicos de qualidade e publicá-los em importantes periódicos são simplesmente admiráveis. Aliar qualidade acadêmica-científica com excelência clínica é uma das características que vêm marcando a trajetória do Dr. Matheus Pithon. Ele possui um consultório particular muito bem-sucedido, em Vitória da Conquista, e é Diplomado pelo Board Brasileiro de Ortodontia (BBO) desde 2011. Um de seus trabalhos recentes, sobre a influência da estética dentária nas chances de se conseguir emprego, mereceu grande destaque no AJO-DO, bem como na grande mídia nacional, sendo motivo de reportagens - como, por exemplo, no Bom Dia Brasil e na Folha de São Paulo. Apesar de todo o sucesso em tão pouco tempo de carreira, o Prof. Pithon é uma pessoa simples, humilde e muito querida por seus alunos. É muito interessante ver a consideração e admiração que acadêmicos do curso de graduação demonstram nos eventos científicos onde o Prof. Matheus os estimula a apresentar trabalhos, tais como os encontros anuais da SBPqO e os bianuais da ABOR. Essas características talvez sejam reflexo dos fortes valores familiares que ele possui. Ele é casado com a dermatologista Ana Carolina, com quem tem um filho de pouco mais de um ano de idade, o João Pedro. Quando não está atendendo no consultório ou escrevendo artigos, ele está curtindo a família e aproveitando sua nova paixão: a criação de gado e cavalos. Nas próximas páginas, o Dr. Matheus Pithon nos conta um pouco sobre sua caminhada na Ortodontia, como ortodontista clínico, pesquisador e professor.

Orthodontic mini-implants (MI) are recommended in different clinical situations and are oftentimes considered as super heroes, especially for recently graduate professionals. In which situations do you recommend the use of MI and what are the major concerns related to their usage? (Antônio Carlos Ruellas)

**Figures 1, 2, 3:**
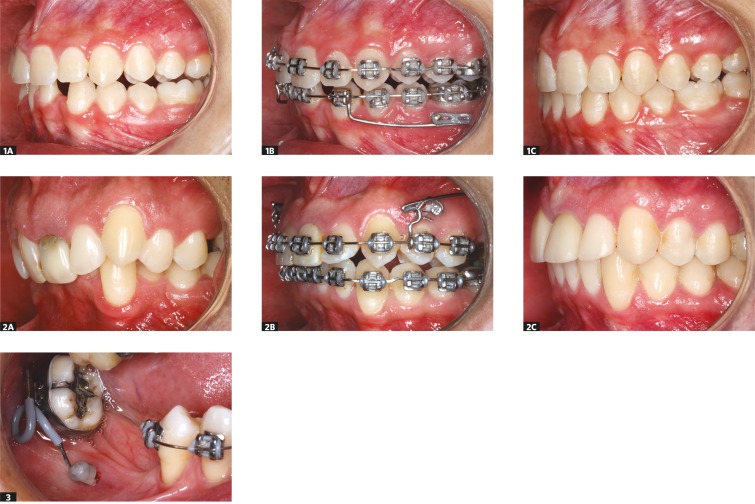
Different mini-implant usages. 1 - Mini-implants used for mesialization of mandibular teeth in a case of agenesis of mandibular lateral incisors: A) initial, B) intermediate (during mesialization performed by means of mini-implant-supported sliding jig), C) after orthodontic treatment finishing. 2 - Mini-implants used to correct Class II malocclusion without extraction - correction was performed by distalization of posterior teeth with the aid of mini-implant-supported sliding jig: A) initial, B) intermediate (during distalization associated with simultaneous intrusion of anterior teeth), and C) finished case. 3 - Uprighting of mandibular molar with significant mesial tipping.

**Figures 4, 5:**
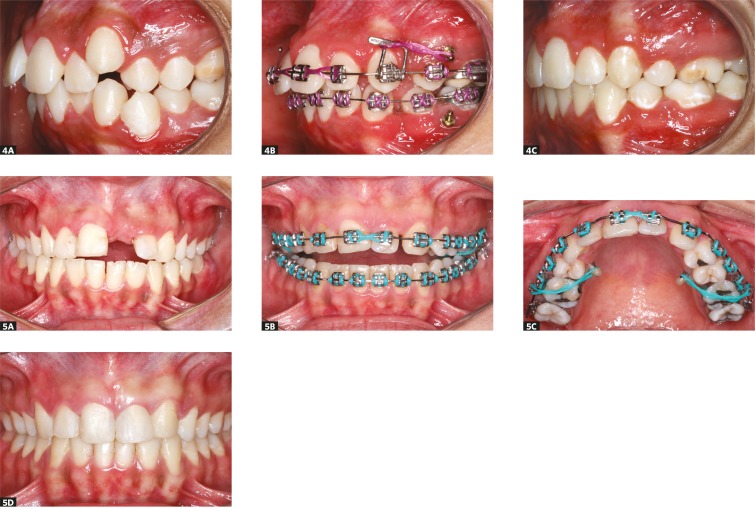
Different mini-implant usages. 4 - Retraction of a maxillary canine: A) initial, B) retraction of the canine performed by means of mini-implant-supported sliding jig, C) finished case. 5 - Intrusion of posterior teeth as an alternative for anterior open bite closure: A) initial, B) intermediate (note moderate open bite), C) occlusal view revealing mini-implants bucally and lingually positioned, D) final (after treatment finishing).

**Figure 6.**
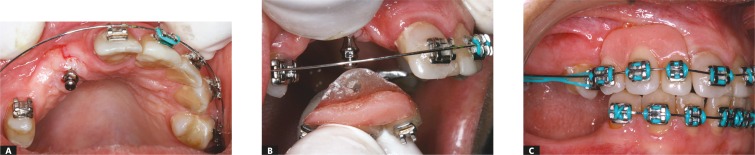
Different mini-implant usages. Mini-implant used as bridge support: A) mini-implant in place, B) bridge fitting to the mini-implant, C) final (after the bridge is fitted over the mini-implant).

Undoubtedly, mini-implants were developed to significantly aid treatment of cases in need of strict orthodontic anchorage control. Nevertheless, rendering them super heroes is rather utopian. Despite being dynamic and advantageous, mini-implants do not work alone, they need to be attached to other devices. That is the heart of the matter; for mini-implants to work properly, the orthodontist has to master orthodontic biomechanics. Precise knowledge of biomechanics is paramount to use mini-implants; should it not be applied, it may lead to failure. A good example is distalization of maxillary teeth performed to correct Class II malocclusion by means of a sliding jig associated with a mini-implant. In these cases, should the sliding jig not be adjusted, it might produce vertical movements (anterior intrusion and posterior extrusion) that hinder final outcomes. In my clinical practice, I often use mini-implants to correct a number of malocclusions: anteroposterior movements along the dental arch (mesialization and distalization), molar uprighting, for supporting retraction of individual teeth, intrusion and some cases of bridge support (Figs 1 to 6). To my view, placing a mini-implant is more practical and fast than bonding an orthodontic accessory to the enamel.

One of the major concerns involved in Orthodontics is related to treatment time. In spite of advances in technology, little has changed. Do you believe skeletal anchorage, particularly that performed by means of mini-implants, contributed to reducing orthodontic treatment time? (David Normando)

Treatment time is the main topic discussed at my office during patient's first visit. Along with orthodontic appliances esthetics, I believe it is a major concern shared by first-time patients. I always talk to patients about it, trying to explain that treatment time is associated with three other factors: (1) severity of the case; (2) biological response; and (3) patient's compliance. This makes patients realize they are also responsible for treatment time. Now, back to your question, I believe skeletal anchorage, particularly that performed by means of mini-implants, has been the greatest revolution Orthodontics has faced in the last 30 years. Mini-implants have allowed treatment to become more predictable and less patient-dependent, at least with regards to anchorage control. As a result, the compliance factor is minimized, thereby reducing total treatment time.

Would you recommend any sequence (or specific elements) of diagnostic procedures that should be considered as priority in order to be able to identify the need for asymmetric extractions in orthodontic treatment? (Antônio Carlos Ruellas) 

**Figure 7.**
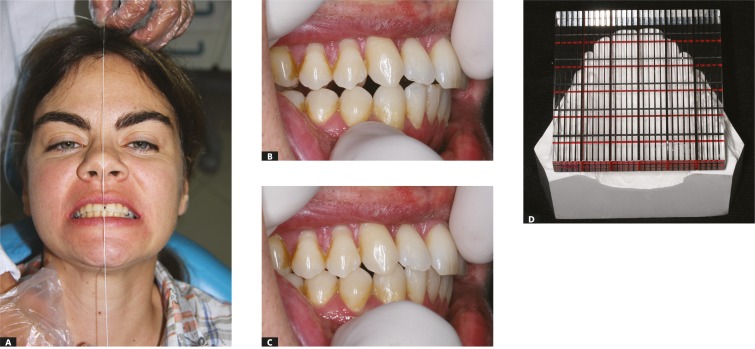
Sequence of diagnostic procedures carried out in cases with dental arch asymmetry: A) checking whether dental midlines coincide with facial midlines, B) patient in maximum intercuspation, C) patient in manually-guided centric relation, and D) assessing anteroposterior asymmetry in dental cast with the aid of a A Schmuth measuring grid.

Orthodontic treatment of asymmetric cases is a daunting challenge to every orthodontist, and an accurate diagnosis is the key to solving these cases. In such situations, I initially assess patient's facial and dental midlines. To this end, I use dental floss going from the Nasion to the Menton and ask patients to carefully move their lips away, with teeth in occlusion (Fig 7A). In frontal view, I can check which midline is deviated. However, assessing function is also necessary, particularly to identify any deflective occlusal contact causing deviation of the mandible (when asymmetries are present in the lower arch) (Figs 7B and C). After assessing midlines and checking whether centric relation coincides with maximal intercuspation, I focus my attention on assessing dental asymmetries in the anteroposterior direction. To this end, I use an orthodontic cast, which I have previously cut, associated with a Schmuth measuring grid (Fig 7D). In some situations, I do not feel satisfied with the orthodontic cast and, for this reason, use a caliper directly into patient's mouth to reach a diagnosis of the problem. I have changed my protocol since mini-implants were introduced as anchorage devices. Depending on the level of asymmetry, I can correct it without the need for extraction. However, this is only possible if patient's tooth-size discrepancy and cephalometric discrepancies are moderate, as well as in the presence of a good facial profile and midline deviation below 4 mm. Should lower arch asymmetry require distalization, it is important to check whether or not there is enough space in the retromolar region.

The use of intraoral anchorage without tooth support caused the number of orthodontic asymmetric extractions to decrease. What were the problems you faced in the past when asymmetric extractions were performed and which you no longer have? (Carlos Flores-Mir)

**Figure 8.**
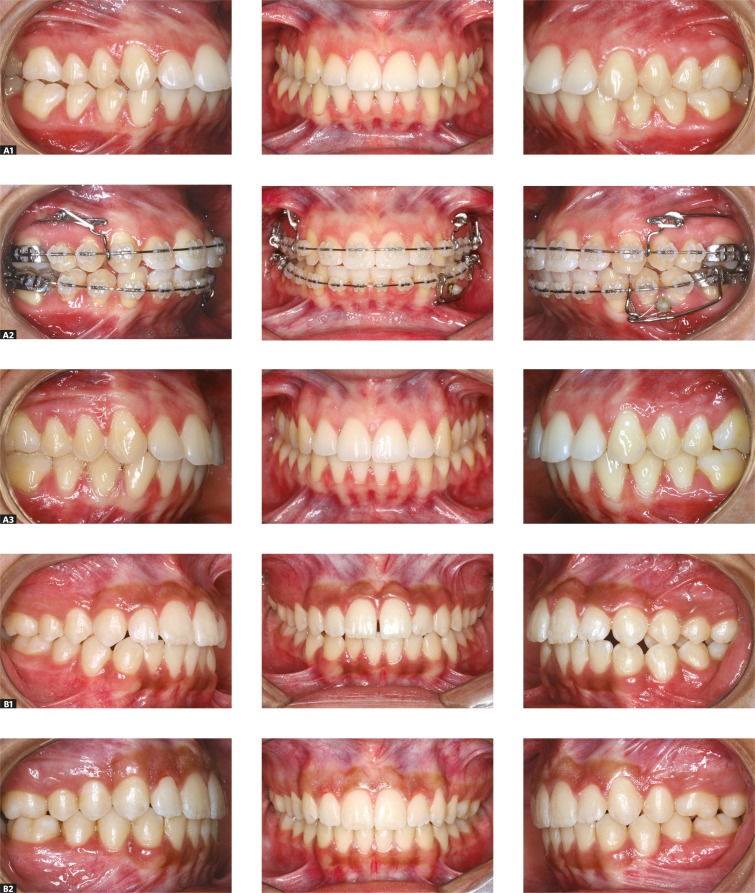
Different clinical cases of asymmetry correction with and without extraction. A1) Initial: 3-mm mandibular asymmetry to the right, in which treatment plan did not include tooth extraction, A2) Intermediate: asymmetry corrected by placing a mandibular mini-implant on one side and two maxillary mini-implants, with a view to correcting dental protrusion, A3) Final: after orthodontic treatment finishing. B1) Initial: 5-mm asymmetry to the right, in which treatment plan included asymmetric extraction of left maxillary premolar, and B2) Final: after debonding of orthodontic appliance.

I do believe that skeletal anchorage performed by means of mini-implants and plates decreases the number of extractions in Orthodontics. Cases that laid on the borderline between extraction and non-extraction undoubtedly benefited from skeletal anchorage. However, extractions remain important and up-to-date, especially for cases requiring dental alignment and leveling, with facial profile maintenance or improvement. As regards asymmetric cases, I believe mini-implants are extremely useful, particularly for cases with asymmetry ranging from 3 to 4 mm. To my view, pronounced asymmetry could be easily corrected by tooth extraction, whether associated or not with mini-implant used as anchorage (Fig 8). The problems I faced in the past when asymmetric extractions were performed and which I no longer have are associated with the best anchorage control provided by mini-implants.

Not rarely, research is in a battle field amidst commercial interests, the need for financial support, conflicts of interest, partnerships and the clinical need for new technology. What is your opinion about these issues? What is your prospect for the future? (Antônio Carlos Ruellas)

Dear Dr. Antônio Carlos, unfortunately, we have to deal with such sad reality. Capitalism, the economic system in which we live, provides us with benefits related to the value of meritocracy and encouragement to work; however, it causes people and companies to compete in a quest for profit and quicker results. In this context, unfair competitors arise. They include ill-intentioned people and companies that disclose or even produce unreal results. Fortunately, parallel to this ongoing conduct there is the growth of means of communication and dissemination of knowledge through the Internet. Thus, new information casting potential doubts can be immediately questioned and checked for authenticity. In Orthodontics, we are going through a delicate moment with regards to self-ligating brackets. Worldwide literature^1^^-^ 4 does not confirm all the advantages disclosed by manufacturers or famous conference speakers. I do believe we need to deepen our studies with a view to speeding up orthodontic treatment; however, at the same time, we should respect patient's individual features and biological response.

Whenever a patient asks you about the possibilities offered by esthetic appliances, what is your answer? What is your esthetic appliance of choice? Do you use esthetic wires? Why? (Dauro Oliveira)

Dr. Dauro, I am a bit traditional when it comes to choosing orthodontic appliances. I strictly follow what I was taught during my undergraduate dental studies. In my office, esthetic appliances are limited to ceramic brackets. This option is always offered to adult patients when they refuse using metal brackets. As for my appliance of choice, I usually opt for polycrystalline brackets with metal slots in cases that need significant tooth movement. Conversely, in cases that need little tooth movement, I opt for monocrystalline brackets, ordinarily known as Sapphire brackets. I use the Edgewise orthodontic technique and, for this reason, need first-order, second-order and third-order bends in the archwires, which makes the use of esthetic wires unfeasible. 

Do you use self-ligating brackets? If so, in which situations? If not, why? (David Normando)

Yes, I do. I believe self-ligating brackets aggregate unique and substantial improvements in comparison to conventional brackets: decreased clinical time necessary for both bracket bonding and orthodontic archwire removal from bracket slots. Unfortunately, orthodontic material manufacturers, increasingly eager to sell, disclose information about this type of material which scientific research published in the best journals around the world cannot prove.^1^^-^ 5 Fortunately, as it happens with any other trend, this is already fading away, as orthodontists who employ the technique in their clinical practice began to realize that the simple mechanism that locks the archwire inside the slot is not enough to modulate biological effects. I wish I had the opportunity to use brackets capable of cutting total treatment time in half and requiring only minor adjustments, as it is widely disclosed. As a supplement to your question, I can say that although I have occasionally opted for self-ligating brackets, I have not used them systematically in my office, particularly because they strongly favor bacterial plaque, when compared to conventional brackets, as proved by two of our recently published studies.^6^^,^ 7

There have not been too many recent advances in what we call "conventional Orthodontics". No major innovations in bracket or wire design can be easily spotted. The attempts to innovate seem to be focused in other processes associated with Orthodontics (mini-implants, corticotomies, vibration, etc.). Why do you think so? (Carlos Flores-Mir)

Dear Dr. Carlos, I believe we have fallen into a state of stagnation as regards the development of new orthodontic material. My view would be that, at this moment in time, we need to make new and radical changes to orthodontic material. In the future, we could think of clear orthodontic wires that could be bent and handled by orthodontists according to each individual case, or bonding material with effective and ongoing fluoride release used to prevent white spot lesions around brackets. However, while these changes are not introduced into our clinical practice, we have to seek scientific evidence to prove the theories about methods used to speed orthodontic movement up, as it is the case of corticotomies, vibration and treatment outcomes yielded by mini-implants and miniplates used as anchorage devices. To my view, we need true thinkers, not mere science replicators.

Do you adopt any methods to speed up/enhance orthodontic movement in your daily clinical practice? Do you believe any method is capable of doing so? (Dauro Oliveira)

Dr. Dauro, to date, I have not used any mechanism to speed up/enhance orthodontic movement in my patients. However, this does not mean I am against that. Nevertheless, I believe science has not stopped evolving in these terms and needs further studies to clarify that matter, particularly clinical trials developed with good methods. Based on systematic literature reviews and meta-analyses, I do believe that corticotomy speeds treatment up;^12^^,^ 13 ^,^ 14 however, I find this procedure rather invasive. In my opinion, advances in research will allow new methods to arise, which will render the procedure simpler and more comfortable to patients.

Not too many orthodontists have an interest in becoming a researcher and professor. As a young man, what motivated you to follow that path and what is the fuel that keeps you interested in this field? (Antônio Carlos Ruellas)

Professor Antônio Carlos, what motivates me is the fact that I can somehow help others; even a tiny new finding, as simple as it might be, will supplement patient's clinical treatment. Unfortunately, in Brazil, science has been performed in mandatory terms. Postgraduate courses have been increasingly pressured to published more and more. As a result, many people are producing science only with a view to reaching a goal. To my view, science should be free, not mandatory. I am not associated with any postgraduate course in Brazil; thus, I do not feel any pressure on my shoulders and I am free to research whatever I want. 

Presently, you are among the group of orthodontists who publish the most worldwide. What advice would you give to those who wish to increase their number of scientific publications? (David Normando)

I believe the best piece of advice I can give to those who wish to increase their number of scientific publications is to love what they do. With the dental market becoming increasingly narrow, many people have pursued an academic career in order to settle down professionally; however, unfortunately, not everybody matches the researcher profile. Conducting new research is hard work and requires not only dedication, but also perseverance, that is the word. I have already had an article of mine refused by 20 journals before it was accepted for publication.^5^Thus, another piece of advice is never giving up publishing your work, even if it has been refused by more than one periodical. Should that be the case, it is important to examine the reviewers' feedback and try to correct your errors. I believe feedback is free-of-charge consulting service.

The number of studies you have published in the last 5 years is highly impressive. What do you consider to be your top three publications in terms of real orthodontic clinical impact? To date, there are several means where we can have research published (peer-reviewed publications - open source or not, books, web based information, etc...). Has this fact changed daily orthodontic clinical practice? (Carlos Flores-Mir)

In the last few years, I have focused my studies on assessing the esthetic perception of changes resulting from orthodontic treatment, as well as on systematic reviews with or without meta-analysis. The first topic was chosen due to the existence of potential doubts on whether or not what orthodontists believe to be esthetic is well accepted by patients. In addition, it is a research process that does not involve high costs and the need for cutting-edge technology. These features also apply to systematic reviews and meta-analyses. Now, in answer to your question, my top three publications are: (1) "Do dental esthetics have any influence on finding a job?"^8 ^This article aimed at assessing whether or not different malocclusions would negatively affect individuals looking for a job. The results of this study revealed that people with an ideal smile have greater chances of being hired as they were considered more intelligent. The group of evaluators comprised people responsible for hiring staff for commercial companies. Importantly, I rank this study as number one in my list because it demonstrates not only the esthetic benefits, but also the social reach of orthodontic treatment. To my surprise and joy, this article was chosen for the cover of October, 2014 issue of American Journal of Orthodontics and Dentofacial Orthopedics. Additionally, I was asked to record a video explaining how the study was conducted and results achieved. Due to being an original article with highly social relevance, I was interviewed by Rede Bahia, a branch of the Brazilian broadcasting TV station Rede Globo. The interview was broadcasted nationally on the news. After that, I was interviewed by two other TV stations, seven magazines and the website UOL. (2) My second most relevant publication was a systematic review entitled "Assessment of the effectiveness of mouthwashes in reducing cariogenic biofilm in orthodontic patients: a systematic review"^9^published in April, 2015 on the Journal of Dentistry. The article addresses a topic of great interest to orthodontists: the prevention of white spot lesions around orthodontic brackets. Many orthodontists advise patients to use mouthwashes to control cariogenic biofilm; however, all of them have the following question in mind: Would mouthwashes really be effective? With a view to answering this question, we conducted this systematic literature review. Results revealed that there is scientific evidence of the effectiveness of mouthwashes in controlling bacterial biofilm. Such results can be clinically applied as an additional tool to prevent the so-feared white spot lesions. (3) My third most relevant publication is the article entitled "Esthetic perception of black spaces between maxillary central incisors by different age groups," also published by AJO-DO.^10^This article aimed at assessing the esthetic perception of laypeople from three different age groups as regards the different levels of black spaces between maxillary central incisors. The idea to conduct this study arose when I noticed the smile of a student of mine who recently had fixed orthodontic appliance removed by an orthodontist, who left a black space between her central incisors. With patient's photograph (at smiling) in hands and with the aid of digital imaging technology, I established different levels of black spaces. Results revealed that black spaces were viewed as negatively scored by all evaluators. Additionally, the younger the evaluator, the worst the score assigned to the black space between central incisors. 

You have been involved in Brazilian clinical practice for a few years. I believe you have already noticed that treatment time reported in Brazil seems to be significantly longer than in other countries. Additionally, it seems to be a set pattern. Why? Can it be considered a problem to patients? Do you foresee short-term changes? (Carlos Flores-Mir)

Undoubtedly, orthodontic treatment time for Brazilian patients is significantly longer than in countries such as USA and Canada. I believe this difference is associated with the economic factor. In Brazil, many patients are treated in research centers that rarely charge for treatment and when they do it is for insignificant amounts. To my view, free treatment neither leads patients to pressure orthodontist to finish the case as fast as possible nor makes orthodontists to feel like doing so. Thus, orthodontists finish their cases according to their own will. Best treatment finishing has been prioritized over treatment time, and this has already been noticed in clinical cases published by Brazilian authors in some international journals, as it is the case of AJO-DO. Additionally, in comparison to private practice in the USA, this has been a common behavior in private Brazilian clinics. In my opinion, the decisive factor here is how treatment is charged in Brazil and overseas. In Brazil, it is common for patients to give a down payment at treatment onset, followed by pre-determined monthly payments until the end of treatment. In the USA, dental treatment is often financed. Thus, the orthodontist receives full payment, which forces him to finish the case as soon as possible so as to welcome a new patient. For this reason, quality is often neglected. To answer your last question, I do not believe this will change in the short-term.

Not rarely, we hear researchers complaining about lack of financial resources for research development, in addition to difficulty in receiving financial support and having their final research product published by prominent journals. You have achieved extraordinary results in the inland of Bahia, which in theory is an underdeveloped region, at a nontraditional university in terms of research. What is the secret of your success? What do you have to say to those who complain about lack of resources and infrastructure? (Dauro Oliveira)

**Figure 9.**
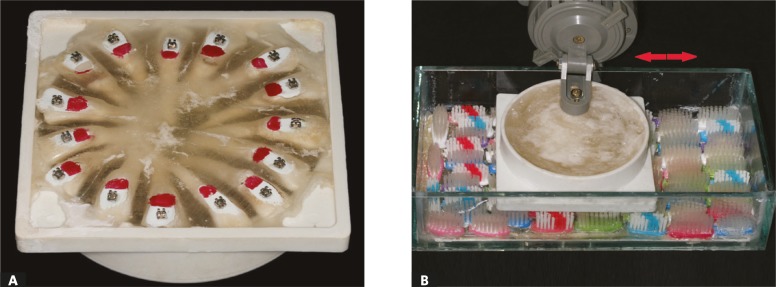
A) Teeth attached to a sink strainer so as to form a single specimen to be taken to the brushing machine; B) Brushing machine comprised by a glass box with tooth brush heads attached to the bottom with hot glue. The engine of a security camera was adapted to perform A B round-tripping movements

Paraphrasing a famous sentence: "The only place where success comes before work is in the dictionary." In other words, those who work hard, focused, with clear goals and love, achieve success. But, in my opinion, there is a lot for me to accomplish before I can say I have achieved success. As regards lack of resources and infrastructure, I am also part of the group clamoring for improvements. Nevertheless, it is useless to keep complaining and doing nothing about it, the problem is huge and out of our control. In Brazil, little attention is given to scientific research, and the career as a scientist is not even regulated by law. Research is carried out by university professors who have to share their teaching, bureaucratic, family and social activities with the lab. However, since I cannot change the reality I live in, I have to seek strategies or bypasses in order to achieve my goal: high-quality scientific research without relying too much on technology and other resources. That is where creativity, inherent to the Brazilian people, comes in: "Make do with what you have." I will give you an example. Two years ago, I had the idea of assessing a new dental varnish that had been recently launched into the market with promises of minimizing the development of white spot lesions around orthodontic brackets. To do so, for the model we had in mind, we would need a machine that brushed teeth in a standardized manner; however, we did not have such technology available. That was when we came up with the idea of designing a brushing machine; and we did, with only US$15,00 (Fig 9). As a result, the study we conducted with the new equipment has been recently published in one of the most important journals of Orthodontics around the world: the European Journal of Orthodontics.^16^ It is worth noting that these publications have allowed me to gain financial support to buy new equipment for our university. Little by little, we have improved our working conditions.

Whenever undergraduate dental students attending Universidade Estadual do Sudoeste da Bahia (UESB) express an interest in Orthodontics, what do you tell them? What is your view of the current dental market? Is it worth being an orthodontist? How do you advise students seeking education in Orthodontics? (Dauro Oliveira)

Dear Dr. Dauro, undergraduates commonly seek advice on which course they should take to study Orthodontics. I always advise them to take up a postgraduate course which has a well-prepared academic staff and is offered with at least 2,000 hours, on a weekly basis, at a higher education institution capable of acknowledging and validating the title. Unfortunately, only the minority listens to me, particularly because the market is full of courses offering an apparent ease: classes taught one weekend per month, lower costs and professors who guarantee to make miracles. In my opinion, the orthodontic market is very good for those who excel in their profession, especially because a high number of patients in need of orthodontic retreatment is indirectly referred to them by unskilled orthodontists who lack appropriate knowledge. For this reason, I claim it is worth being an orthodontist; however, I emphasize the need for Orthodontists with a capital "O".
